# Supplementary material for: Cultivation potential of the tropical carrageenophyte Eucheumatopsis isiformis (Solieriaceae, Rhodophyta) from Yucatán, Mexico
Source: PLoS One. 2026 Apr 22;21(4):e0346826. doi: 10.1371/journal.pone.0346826 (PMC13102206; doi:10.1371/journal.pone.0346826)
Supplement: S1 File — Molecular analysis. Coding, date of collection and haplotype description used for construction of the Bayesian phylogram of Eucheumatopsis from Yucatán, Mexico. Haplotype description is based on gene sequencing of cox1, rbcL, and RuBisCo spacer. S2 Table. Carrageenan content. Native and alkali-treated carrageenan yields and sulfate content of Eucheumatopsis isiformis for the different morphotypes collected from March to November 2022. S3 Table. Morphological and anatomical characterization. Morphological characteristics of Eucheumatopsis isiformis morphotypes. Comparisons with specimen from Florida, United States of America (USA). S4 Fig. Morphological characterization. Specimen identified as Eucheumatopsis isiformis from Bahia Honda, Florida, USA. (ZIP) [file pone.0346826.s001.zip › S1_Table.pdf]

S1 Table. Molecular analysis. Coding, date of collection and haplotype description used for construction of the Bayesian phylogram of *Eucheumatopsis* from Yucatán, Mexico. Haplotype description is based on gene sequencing of *cox*1, *rbc*L, and RuBisCo spacer.

Number	Morphotype	Date	Locality	Herbarium	Label
1	M1	14-Sep-23	Dzilam de Bravo		*Eucheumatopsis isiformis* morphotype 1 (September 14, 2023) M1
2	M3	14-Sep-23	Dzilam de Bravo		*Eucheumatopsis isiformis* morphotype 3 (September 14, 2023) 2M3
3	M1	24-Oct-23	Dzilam de Bravo	*	*Eucheumatopsis isiformis* morphotype 1 (October 24, 2023)1M1
4	M2	24-Oct-23	Dzilam de Bravo	*	*Eucheumatopsis isiformis* morphotype 2 (October 24, 2023) 3M2
5	M3	24-Oct-23	Marine Station	*	*Eucheumatopsis isiformis* morphotype 3 (October 24, 2023) 5M3D
6	M3	24-Oct-23	Marine Station		*Eucheumatopsis isiformis* morphotype 3 (October 24, 2023) 7M3
7	M3	24-Oct-23	Laboratory	*	*Eucheumatopsis isiformis* morphotype 3 (October 24, 2023) 6M3
8	M2	08-Dec-23	Telchac	*	*Eucheumatopsis isiformis* morphotype 2 (December 08, 2023) 8M2
9	M3	08-Dec-23	Telchac	*	*Eucheumatopsis isiformis* morphotype 3 (December 08, 2023) 9M3
10	M2	08-Dec-23	Telchac	*	*Eucheumatopsis isiformis* morphotype 2 (December 08, 2023) 10M2T
11	M1	08-Dec-23	Telchac	*	*Eucheumatopsis isiformis* morphotype 1 (December 08, 2023) 11M1T
12	M2	22-Mar-24	Dzilam de Bravo	*	*Eucheumatopsis isiformis* morphotype 2 (March 22, 2024) 1M2
13	M2	22-Mar-24	Dzilam de Bravo	*	*Eucheumatopsis isiformis* morphotype 2 (March 22, 2024) 2M2
14	M3	22-Mar-24	Dzilam de Bravo	*	*Eucheumatopsis isiformis* morphotype 3 (March 22, 2024) 3M3
15	M2	22-Mar-24	Dzilam de Bravo	*	*Eucheumatopsis isiformis* morphotype 2 (March 22, 2024) 4M2
16	M3	22-Mar-24	Dzilam de Bravo		*Eucheumatopsis isiformis* morphotype 3 (March 22, 2024) 5M3
17	M2	22-Mar-24	Dzilam de Bravo	*	*Eucheumatopsis isiformis* morphotype 2 (March 22, 2024) 6M2
18	M2	22-Mar-24	Dzilam de Bravo	*	*Eucheumatopsis isiformis* morphotype 2 (March 22, 2024) 7M2
19	M2	22-Mar-24	Dzilam de Bravo	*	*Eucheumatopsis isiformis* morphotype 2 (March 22, 2024) 8M2
20	M2	22-Mar-24	Dzilam de Bravo	*	*Eucheumatopsis isiformis* morphotype 2 (March 22, 2024) 9M2
21	M3	22-Mar-24	Dzilam de Bravo	*	*Eucheumatopsis isiformis* morphotype 3 (March 22, 2024) 10M3
22	M2	22-Mar-24	Dzilam de Bravo	*	*Eucheumatopsis isiformis* morphotype 2 (March 22, 2024) 11M2
23	M2	22-Mar-24	Dzilam de Bravo	*	*Eucheumatopsis isiformis* morphotype 2 (March 22, 2024)12M2
24	M2	22-Mar-24	Dzilam de Bravo	*	*Eucheumatopsis isiformis* morphotype 2 (March 22, 2024) 13M2
25	M1	22-Mar-24	Dzilam de Bravo	*	*Eucheumatopsis isiformis* morphotype 1 (March 22, 2024) 14M1
26	M1	22-Mar-24	Dzilam de Bravo	*	*Eucheumatopsis isiformis* morphotype 1 (March 22, 2024) 15M1
27	M1	22-Mar-24	Dzilam de Bravo	*	*Eucheumatopsis isiformis* morphotype 1 (March 22, 2024) 16M1
28	-	08-Apr-24	Laboratory		*Kappaphycus* LabMEX

Haplotype description is based on gene sequencing of *cox*1, *rbc*L, and RuBisCo spacer.

**Haplotype**	**Nucleotide states at variable sites*** 1122478991 0637188442 2876737252 8	**n**	**Sample**
Hap01	AAATATAGAT	22	10M3, 2M2, 12M2, 8M2, 2M3, 10M2T, 1M1, 7M2, 11M1T, 3M2, 6M3, 4M2, 5M3D, 15M1, 1M2, 16M1, 5M3, 6M2, 14M1, 9M2, 7M3, 8M2
Hap02	AAACATAGAT	5	11M2, 9M3, M1, 13M2, 3M3
Hap03	AAATACTAGC	1	*Eucheuma isiforme*
Hap04	GGGCGTAGAT	1	*Eucheumatopsis isiformis*

*Nucleotide composition at polymorphic positions in the concatenated *cox*1–*rbc*L–RuBisCo spacer alignment. GenBank: *Eucheuma isiforme* AF099691 (*rbc*L); *Eucheumatopsis isiformis* MG948344 (*rbc*L) - MG948345 (*cox*1).
