# Supplementary material for: Cultivation potential of the tropical carrageenophyte Eucheumatopsis isiformis (Solieriaceae, Rhodophyta) from Yucatán, Mexico
Source: PLoS One. 2026 Apr 22;21(4):e0346826. doi: 10.1371/journal.pone.0346826 (PMC13102206; doi:10.1371/journal.pone.0346826)
Supplement: S1 File — Molecular analysis. Coding, date of collection and haplotype description used for construction of the Bayesian phylogram of Eucheumatopsis from Yucatán, Mexico. Haplotype description is based on gene sequencing of cox1, rbcL, and RuBisCo spacer. S2 Table. Carrageenan content. Native and alkali-treated carrageenan yields and sulfate content of Eucheumatopsis isiformis for the different morphotypes collected from March to November 2022. S3 Table. Morphological and anatomical characterization. Morphological characteristics of Eucheumatopsis isiformis morphotypes. Comparisons with specimen from Florida, United States of America (USA). S4 Fig. Morphological characterization. Specimen identified as Eucheumatopsis isiformis from Bahia Honda, Florida, USA. (ZIP) [file pone.0346826.s001.zip › S2_Table.pdf]

S2 Table. Carrageenan content. Native and alkali-treated carrageenan yields and sulfate content of *Eucheumatopsis isiformis* for the different morphotypes collected from March to November 2022.

		**Native**		**Alkali treated**	
E. isiformis	**Collection date**	**Yield (%)**	**Sulfate (%)**	**Yield (%)**	**Sulfate (%)**
Morphotype 1	March	46.3 ± 0.1	30.3 ± 1.9	40.1 ± 0.1	27.3 ± 1.7
	May	52.3 ± 1.5	32.0 ± 1.6	38.1 ± 1.2	26.1 ± 1.3
	July	57.4 ± 0.9	22.4 ± 1.5	46.3 ± 0.8*	21.9 ± 1.5
	September	46.0 ± 0.1	29.1 ± 0.2	46.3 ± 2.6	22.7 ± 1.1
	November	46.3 ± 0.9	36.0 ± 0.5	35.3 ± 1.1	30.5 ± 1.6
Morphotype 2	March	39.3 ± 1.3	32.6 ± 0.3	-	-
	May	53.6 ± 1.8	31.7 ± 0.4	35.9 ± 2.0	26.0 ± 1.1
	July	-	-	-	-
	September	-	-	-	-
	November	-	-	-	-
Morphotype 3	March	46.1 ± 0.1	30.5 ± 1.2	43.2 ± 1.5	25.4 ± 0.9
	May	42.9 ± 1.2	31.2 ± 0.9	43.2 ± 0.8	24.6 ± 1.3
	July	58.0 ± 1.2	32.7 ± 1.0	39.0 ± 0.3	23.6 ± 0.7
	September	-	-	-	-
	November	-	-	-	-

Mean values with ± standard error of the mean is shown ($n \geq 3$) * indicates a statistically significant difference with $p \leq 0.01$ and - indicates no specimens were found.
