# Supplementary material for: Cultivation potential of the tropical carrageenophyte Eucheumatopsis isiformis (Solieriaceae, Rhodophyta) from Yucatán, Mexico
Source: PLoS One. 2026 Apr 22;21(4):e0346826. doi: 10.1371/journal.pone.0346826 (PMC13102206; doi:10.1371/journal.pone.0346826)
Supplement: S1 File — Molecular analysis. Coding, date of collection and haplotype description used for construction of the Bayesian phylogram of Eucheumatopsis from Yucatán, Mexico. Haplotype description is based on gene sequencing of cox1, rbcL, and RuBisCo spacer. S2 Table. Carrageenan content. Native and alkali-treated carrageenan yields and sulfate content of Eucheumatopsis isiformis for the different morphotypes collected from March to November 2022. S3 Table. Morphological and anatomical characterization. Morphological characteristics of Eucheumatopsis isiformis morphotypes. Comparisons with specimen from Florida, United States of America (USA). S4 Fig. Morphological characterization. Specimen identified as Eucheumatopsis isiformis from Bahia Honda, Florida, USA. (ZIP) [file pone.0346826.s001.zip › S3_Table.pdf]

S3 Table. Morphological and anatomical characterization. Morphological characteristics of *Eucheumatopsis isiformis* morphotypes. Comparisons with specimen from Florida, United States of America (USA).

	**Yucatán, Mexico**			**Florida, USA**
	**Morphotype 1**	**Morphotype 2**	**Morphotype 3**	
Thallus height (cm)	28 - 38	23 - 47	Up to 32	Up to 20
Branching	Irregular, scarce, up to 3- 4 orders	Irregular, abundant in distal areas, up to 5 orders	Irregular, up to 3 orders, rarely 4 orders	Irregular, up to 3 orders
Branches	Main axis evident, slender, cylindrical, smooth or with upcurved spines scattered all over the thallus, small branchlets on the 3rd order branches at distal areas, branches constricted at the bases	Cylindrical, abundant spines and small branchlets scattered in distal areas; branches constricted at the bases. Axes smooth, without spines at basal areas	Main axis evident, thick, cylindrical, abundant up-curved spines scattered all over the thallus, some spines at 90° angles respect to the thallus, scarce small branchlets, branches slightly constricted at the bases	Cylindrical, abundant upcurved spines scattered all over the thallus or at 90° angles with respect to the thallus. Without constriction at the bases of the branches
Spines	Simple, upcurved	Simple or divide	Simple, upcurved or straight, some divided	Simple, upcurved or straight
Apex	Acute, some truncated with regrowth	Round to acute, some forked	Acute, some truncated with regrowth	Acute, some forked

Branches diameter (mm)	3 - 3.5, ≤ 1 at the bases	1 - 4, 1 at the bases	2 - 4.5, 1 -2 at the bases	1 - 2
Branchlets or spines length (mm)	0.1 - 2	0.1 - 1.8	0.1 - 1.2	NA
Reproductive structures	Cystocarp on small branchlets in the main axis	Cystocarp at the base of small branchlets in the main axis	Cystocarp at the base of small branchlets in the main axis	NA

NA indicates no available information. For quantitative data, *n*=50.
