# Supplementary material for: Cultivation potential of the tropical carrageenophyte Eucheumatopsis isiformis (Solieriaceae, Rhodophyta) from Yucatán, Mexico
Source: PLoS One. 2026 Apr 22;21(4):e0346826. doi: 10.1371/journal.pone.0346826 (PMC13102206; doi:10.1371/journal.pone.0346826)
Supplement: Attachment — Submitted filename: renamed_2b074.docx [file pone.0346826.s002.docx]

**Subject:** Reviewer’s Comments on Manuscript PONE-D-25-36873

**To:** The Editor, *PLOS ONE*

Dear Editor,

I have carefully reviewed the manuscript entitled

**“Studies on the tropical carrageenophyte Eucheumatopsis isiformis (Solieriaceae, Rhodophyta) from Yucatán, Mexico: cultivation potential based on morphological, chemical, and reproductive features.”** This manuscript presents valuable baseline information on the reproductive biology, biochemical composition, and cultivation potential of the red alga *Eucheumatopsis isiformis* collected from Yucatán, Mexico. The topic is both relevant and timely, as it contributes to the understanding of native *eucheumatoid* species and their potential applications in sustainable aquaculture, an area of increasing scientific and economic importance.

The study integrates multiple aspects including field sampling, morpho-anatomical characterization, molecular analysis, and carrageenan composition, offering a comprehensive foundation for further research on *E. isiformis*.

However, while the study is scientifically significant, the manuscript requires structural refinement, stronger statistical justification, and improved consistency in data presentation to meet publication standards. My detailed comments are summarized below for your consideration.

**Major Comments**

**1. Lack of variability reporting in polyamine-induced sporulation experiment**

In the results of the polyamine-induced sporulation experiment (Page 378–382; Fig. 6), only the carpospore counts are presented (e.g., “363 carpospores per 5 mL…”), without reporting standard deviation (SD) values or error bars in the figure. Although the “Materials and Methods” section (Page 10, Lines 195–199) indicates that each treatment was performed in triplicate (n = 3), this information is not reflected in the results. The authors should include mean ± SD (or SE) values and, if applicable, significance indicators among treatments to ensure statistical completeness and data reliability.

**2. Unclear justification for the use of non-parametric tests**

The manuscript applies both parametric (ANOVA) and non-parametric (Kruskal–Wallis) tests, but the justification for using the non-parametric approach is unclear. Although the Statistical Analysis section (Page 10–11, Lines 208–221) mentions the use of Shapiro–Wilk and Levene’s tests to assess normality and homogeneity, the authors do not specify which dataset(s) failed these assumptions. Please clarify which data did not meet normality, report corresponding test results (e.g., *p* < 0.05), and explain the rationale for applying non-parametric analysis.

**Minor Comments**

1) **Salinity units:**

The unit of salinity should be expressed consistently throughout the manuscript according to the instrument used for measurement. Please use **PSU (Practical Salinity Unit)** or **ppt (parts per thousand)** instead of **ppm**, which is not appropriate for salinity reporting in marine studies.

2) **Incomplete references:**

Some references are missing publication years (e.g., “Refs. [37–38, 62–66]”). Please check all references carefully and complete missing bibliographic information in accordance with the journal’s reference style.

3) **Statistical notation consistency:**

Ensure consistent formatting of statistical terms (e.g., *n = 3*, *p* < 0.05, mean ± SD) throughout the manuscript, tables, and figure captions.

4) **Figure legends:**

Include the number of replicates (*n*) and clarify whether values are presented as mean or median in all figure legends.

5) **Abbreviation clarity:**

Check that all abbreviations (e.g., SPE, PUT, PES) are defined at first mention in both the text and figure captions.

In summary, the manuscript is scientifically promising and provides useful data for future studies on the reproductive biology and mariculture potential of *E. isiformis*. However, revisions are necessary to improve clarity, statistical transparency, and some issues that require correction before it can be considered for publication.

Thank you for the opportunity to review this work. I hope these comments will assist the authors in refining their manuscript and contribute to improving the overall quality of the publication.

Sincerely,
