# Supplementary material for: Cultivation potential of the tropical carrageenophyte Eucheumatopsis isiformis (Solieriaceae, Rhodophyta) from Yucatán, Mexico
Source: PLoS One. 2026 Apr 22;21(4):e0346826. doi: 10.1371/journal.pone.0346826 (PMC13102206; doi:10.1371/journal.pone.0346826)
Supplement: Attachment — Submitted filename: Review-Final.docx [file pone.0346826.s003.docx]

**Studies on the tropical carrageenophyte Eucheumatopsis isiformis (Solieriaceae, Rhodophyta) from Yucatán, Mexico: cultivation potential based on morphological, chemical, and reproductive features”**

1. Title is **scientifically sound and informative**, but it is **quite long and descriptive**.

May be considered

**Cultivation potential of the tropical carrageenophyte *Eucheumatopsis isiformis* (Solieriaceae, Rhodophyta) from Yucatán, Mexico**

2. The abstract effectively highlights the cultivation potential of *Eucheumatopsis isiformis*; it could be improved by briefly summarizing the key morphological and molecular differences among the analyzed morphotypes to strengthen the connection between diversity and cultivation potential.
3. The introduction is too lengthy, which may overwhelm readers.
4. Some background information especially on general seaweed life cycles and previously studied *Eucheuma* species—could be condensed to maintain focus on *Eucheumatopsis isiformis* and the specific research gap.
5. Although the section includes relevant citations up to 2022, it lacks very recent studies (2023–2024) on seaweed genetic improvement, reproductive biology, or carrageenan yield optimization. Including these would enhance the currency and scientific depth of the background.
6. The introduction should more clearly highlight why *E. isiformis* was chosen, how it differs from commercial Eucheuma species, and **what specific research questions or hypotheses this study addresses.**
7. The section is well-structured and provides comprehensive methodological details for replication. Provide subheadings with clearer separation (e.g., Sample processing, Microscopy and staining, Data analysis) to enhance readability, as the current format is text-heavy and occasionally dense.
8. The sporulation induction and carrageenan extraction procedures are well explained, but the rationale for selecting specific concentrations of polyamines and irradiance levels could be briefly justified with relevant citations or preliminary data to strengthen methodological reasoning.
9. The section demonstrates appropriate use of statistical tests; however, the choice of parametric vs. non-parametric methods could be better justified. Additionally, information on software used for analysis (e.g., SPSS, R, or GraphPad) should be explicitly mentioned for transparency and reproducibility.
10. Some sentences are overly long and could be simplified. There are minor typographical errors (e.g., double periods in “PCR conditions.” and “residual Cetavlon.”). Consistent use of units (e.g., “μmol·m⁻²·s⁻¹”) and spacing around symbols should be checked for formatting accuracy.
11. The result section provides rich data but could benefit from clearer subheadings for each main result (e.g., Morphoanatomical characterization, Carrageenan content, Molecular analysis, Reproductive phases, Sporulation experiments). This would enhance readability and help the reader follow the logical sequence of findings.
12. The text contains excessive numeric detail (e.g., every mean and standard deviation is listed in sentences as well as in tables). This makes the narrative dense and repetitive. Consider focusing the text on key trends, comparisons, and significant findings, while leaving raw data to tables and supplementary files.
13. The integration of figures and tables is appropriate, but figure legends and table references could be clearer. Some figure legends (e.g., Figs. 6–7) could briefly summarize the key outcome (e.g., “Carpospore release enhanced under 10⁻³ M PUT treatment compared with control”).
14. While some statistical results (e.g., F=9.57, p<0.01; H=75.74, p<0.01) are reported, interpretation of their biological meaning is limited. It would strengthen the result section to discuss what these differences imply e.g., whether morphotype differences correlate with environmental adaptation, or whether the yield reduction post-alkaline treatment is significant for industrial use.
15. The molecular analysis is concise and clear, but lacks a visual reference (e.g., phylogenetic tree figure or haplotype network). The paragraph would be stronger if you explicitly noted whether the lack of genetic differentiation despite morphological variability suggests phenotypic plasticity or cryptic speciation.
16. The experiment is well-detailed, but results and interpretation are somewhat blended with methods. For instance, repeated mentions of acclimatization periods, temperature, and irradiance fit better in the Methods. Here, focus on outcome patterns e.g., “Putrescine (10⁻³ M) significantly enhanced carpospore release at lower irradiance, although variability across days was noted” to clarify the biological relevance.
17. The discussion contains a lot of detailed data and literature references, which demonstrate strong experimental support. However, it would benefit from more concise synthesis clearly highlighting how your findings advance understanding of *E. isiformis* biology or cultivation compared to previous studies. Some paragraphs could be shortened by summarizing numerical data instead of repeating all values.
18. While the text notes that no genetic differences were observed among morphotypes, it could be strengthened by discussing the implications of this finding for example, whether this morphological variability is due to environmental plasticity or phenotypic adaptation.
19. The discussion ends with good experimental observations on polyamine-induced sporulation, but it should also clearly state the broader implications such as how these findings contribute to large-scale cultivation strategies or seedbank development for tropical carrageenophytes.
20. Adding 2–3 lines on how this work supports future selective breeding or aquaculture resilience would make the conclusion stronger and more impactful.
21. The conclusion clearly outlines future research directions but could be more concise and focused on the main findings such as species identification, morphotype characterization, and successful sporulation induction before discussing future perspectives.
22. It would be beneficial to separate experimental details from general recommendations, presenting a clearer summary of key outcomes followed by suggested future studies for better readability and impact.
